# Retraction: Identification of Autoantibodies against Transthyretin for the Screening and Diagnosis of Rheumatoid Arthritis

**DOI:** 10.1371/journal.pone.0205914

**Published:** 2018-10-11

**Authors:** The PLOS ONE Editors

After publication of this article [1], concerns were raised about the Western blot and immunoprecipitation/Western blot data reported in Figs 2, 3, 4, 6 and 8:

- Lanes 4–7 of the blot shown in Fig 4 appear highly similar to lanes 4–7 of the blot in Fig 8. Also, lanes 8–9 in the Fig 8 blot are highly similar to lanes 4–5 of the blots in Figs 4 and 8. Figs 4 and 8 represent different experiments, and the bands in question reflect data for different patients and/or experimental groups.
- The brightness and contrast in Figs 2, 3, 4, 6, and 8 are adjusted such that one cannot confirm the integrity of the blot images.
- The Western blot experiments in Figs 2, 3, 4, and 6 did not include loading controls, such as accompanying blots or stripped experimental blots that are probed with an antibody to a standard housekeeping gene.
- The immunoprecipitation/Western blot experiment in Fig 8 did not include a control to demonstrate the specificity of the immunoprecipitation, such as a sample immunoprecipitated with a non-specific primary antibody or a sample for which a primary antibody had not been added.
- The quantification data shown in the figures reflected Western blot data obtained across multiple blots that are based on ECL detection of proteins and furthermore were not normalized.

We discussed these concerns with the authors, who noted that the original raw data underlying Figs 2, 3, 4, 6, and 8 are unavailable. They replicated the experiments in question, but again did not include appropriate controls. While the replication data support most results as reported in the original figures, we are concerned about the experimental data, in which protein levels are compared across samples and across blots without normalizing loading controls. The concerns about the Western blot experiments as well as about potential image duplications in Figs 4 and 8 call into question the validity of the claims made.

In light of these issues, the *PLOS ONE* Editors retract this article. We apologize that the concerns about experimental controls and data normalization were not raised during the pre-publication review process.

SS agreed with the retraction. SG, AS, RM, OPG, YS, RSS, DSB, and TKD did not respond. LKS could not be reached. SB did not agree with retraction.
